# Platelet-mitochondria dual-targeted nanocarriers for enhanced empagliflozin therapy in atherosclerosis

**DOI:** 10.3389/fbioe.2025.1638034

**Published:** 2025-09-08

**Authors:** Yue Tang, Yue Tian, Yi Wang, Xue Mei, Lijun Luo, Fan Zhang, XiaoJin Gao, Yihua Wang, Jun Hou, Chunyang Zhou

**Affiliations:** ^1^ Institute of Materia Medica, School of Pharmacy, North Sichuan Medical College, Nanchong, China; ^2^ Department of Cardiology, The third People’s Hospital of Chengdu, Chengdu, China; ^3^ School of Life Science and Engineering, Southwest Jiaotong University, Chengdu, China; ^4^ Institute of Hepato-Biliary-Pancreatic-Intestinal Disease, Affiliated Hospital of North Sichuan Medical College, Nanchong, China

**Keywords:** atherosclerosis, empagliflozin, platelet membrane-cloaked nanoparticles, targeted delivery, mitochondria-targeted

## Abstract

**Introduction:**

Atherosclerosis (AS) is a primary cause of cardiovascular disease and significantly contributes to the global disease burden. Empagliflozin (EMP), a candidate drug for AS treatment, has not been clinically approved due to challenges including poor solubility, low bioavailability, and potential toxicity.

**Methods:**

To address these challenges, we constructed a platelet membrane-biomimetic, mitochondria-targeted delivery system (PM@EPPT). This system was developed by loading EMP into PCL-PEG polymeric micelle, modifying the PEG terminus with triphenylphosphine (TPP), and coating the nanoparticle surface with platelet membranes. We then evaluated its efficacy against AS using both *in vitro* and *in vivo* models.

**Results:**

The PM@EPPT system exhibited favorable physical properties and biocompatibility. *In vitro*, it alleviated oxidative stress-induced macrophage apoptosis by scavenging reactive oxygen species (ROS), restoring mitochondrial membrane potential, and activating mitophagy. In ApoE^-/-^ mouse models, PM@EPPT significantly reduced aortic plaque area by 43%, decreased the expression of inflammatory markers (CD68 and MMP-9), increased levels of the plaque stability marker (α-SMA), and improved lipid profiles.

**Discussion:**

In conclusion, PM@EPPT enhances EMP bioavailability through platelet membrane-mediated arterial plaque targeting and TPP-modified mitochondrial targeting. This study provides experimental evidence for optimizing EMP efficacy in AS treatment and developing therapeutic platforms for other poorly soluble drugs targeting AS.

## Introduction

Cardiovascular diseases (CVDs) are the primary causes of disability and mortality worldwide, affecting over 523 million people. Alarmingly, the number of CVD-related deaths is projected to rise to 35.6 million by 2050, representing a 73.4% surge in crude mortality compared to 2025 (Chong et al., 2024; Nedkoff et al., 2023; Vollset et al., 2024). Atherosclerosis (AS), the core pathological basis of most CVDs, is characterized by abnormal lipid accumulation in arterial walls and persistent chronic inflammatory responses, ultimately leading to clinical events such as myocardial infarction and stroke. Current AS treatments primarily rely on lifestyle interventions and lipid-lowering strategies with statins. However, these approaches have limitations in halting plaque progression, especially regarding precise intervention in the complex pathological processes within vulnerable plaques (e.g., local inflammation, oxidative stress). This results in significant residual cardiovascular risk (Kumric et al., 2023; Björkegren and Lusis, 2022).

Empagliflozin (EMP), a sodium-glucose cotransporter 2 inhibitor (SGLT2i), has demonstrated cardioprotective benefits independent of glycemic control in multiple large clinical trials, significantly reducing the risk of heart failure hospitalization and cardiovascular death. Basic research shows that EMP delays AS progression through multiple mechanisms, including regulating lipid metabolism, improving endothelial function, inhibiting inflammatory responses, and crucially, enhancing mitochondrial function (Fitchett et al., 2020; Hernandez et al., 2024; Gaborit et al., 2021; Liu Z. et al., 2021). However, as a Biopharmaceutics Classification System (BCS) Class II drug, EMP has inherently low solubility (0.11 mg/mL), undergoes rapid *in vivo* clearance, and exhibits non-specific distribution. These properties lead to low bioavailability and potential systemic side effects such as genital infections and ketoacidosis, severely limiting the potential of EMP in treating AS (Biondi-Zocca et al., 2024; Yadav et al., 2024).

Nanodrug delivery systems (NDDS) offer new ideas for improving EMP delivery. Nanocarriers can significantly enhance the bioavailability of poorly soluble drugs by increasing the specific surface area and altering dissolution kinetics. Biocompatible polymeric materials (e.g., PEG, PCL, PLGA) are ideal carrier choices due to their controllable degradability and easy functional modification (Liu et al., 2023; Mao et al., 2023; Mahmood et al., 2023; Singh, 2024). However, traditional nanoparticles are rapidly cleared by the mononuclear phagocyte system (MPS) during systemic circulation and lack specific targeting ability toward AS vulnerable plaques. Given EMP’s multi-target mechanism, achieving drug enrichment specifically at lesions is crucial to maximize efficacy and minimize off-target toxicity (Raza et al., 2024; Behl et al., 2023; Sreena and Nathanael, 2023; Zinger, 2023).

In recent years, biomimetic nanotechnology based on natural cell membranes has provided a new path to solve the targeting bottleneck (Zinger, 2023; An et al., 2025; Ijaz et al., 2024; Zhang et al., 2024). Among them, platelet membrane (PM)-coated nanocarriers have shown significant advantages in AS treatment due to their unique pathological site homing ability: (1) PM coating enables carriers to “inherit” the active targeting ability of activated platelets to damaged endothelium; (2) surface proteins on PM (such as CD47) endow carriers with excellent immune evasion properties, prolonging the circulation half-life; and (3) significantly improved drug accumulation efficiency in plaques Meanwhile, mitochondrial dysfunction (manifested as membrane potential collapse, ROS burst, and impaired autophagy) is a core link driving AS progression. Although EMP can effectively improve mitochondrial function, conventional delivery systems still cannot achieve efficient drug delivery to mitochondria (Jiang et al., 2024; Jan et al., 2024; Safdar et al., 2024).

Given the central role of mitochondria in AS pathology and the action dependency of EMP, achieving subcellular organelle targeting is crucial. Healthy mitochondria maintain a high transmembrane negative potential (ΔΨ_m_ = −150 to −180 mV) through the electron transport chain. Triphenylphosphine (TPP), a lipophilic cationic compound, can actively utilize the electrochemical gradient driven by ΔΨ_m_ to achieve 10–100 times higher drug concentration enrichment in the mitochondrial matrix than in the cytoplasm (Nardin et al., 2023; Martinez Bravo et al., 2024; Ba et al., 2024).

To overcome the pharmacokinetic defects of EMP and achieve efficient delivery to the lesion and mitochondrial core targets, this study constructed a platelet membrane biomimetic and mitochondria-targeted delivery system (PM@EPPT). This system integrates three functional modules: (1) a PCL-PEG polymeric micelle that efficiently encapsulates EMP and improves solubility; (2) TPP surface modification that utilizes ΔΨ_m_ to drive precise accumulation of drugs in mitochondria; (3) platelet membrane coating that endows carriers with active lesion-targeting ability and long-circulating properties. This dual design, through phased delivery (targeting plaque tissues first and then diseased organelles), is expected to intervene more effectively in key pathological processes of atherosclerosis - such as oxidative stress and apoptotic disorders caused by mitochondrial dysfunction.

## Materials and methods

### Materials

Polyethylene glycol (PEG 2000), ε-caprolactone, 3-bromo-1-propanol (CAS 627–18–9), triphenylphosphine, 1-ethyl-3-(3-dimethylaminopropyl)carbodiimide hydrochloride (EDC·HCl), N,N-dimethylformamide (DMF), and N-hydroxysuccinimide (NHS) were purchased from Sigma-Aldrich (St. Louis, MO, United States of America). Empagliflozin (EMP), prostaglandin E_1_ (PGE_1_), ethylenediaminetetraacetic acid (EDTA) and Sulfo-Cyanine5.5(Cy5)were obtained from MedChemExpress (Monmouth Junction, NJ, United States of America). Annexin V-fluorescein isothiocyanate (Annexin V-FITC) and Dulbecco’s modified Eagle’s medium (DMEM) were sourced from Solarbio (Beijing, China). Calcein-AM/PI, JC-1 (5,5′,6,6′-tetrachloro-1,1′,3,3′-tetraethylbenzimidazolylcarbocyanine iodide), and 4′,6-diamidino-2-phenylindole (DAPI) were acquired from Beyotime Biotechnology (Shanghai, China). MitoTracker Green FM and CellROX Deep Red reagent were purchased from Thermo Fisher Scientific (Waltham, MA, United States of America). Fetal bovine serum (FBS) was obtained from Thermo Fisher Scientific. Oxidized low-density lipoprotein (ox-LDL, 50 μg/mL, endotoxin <0.1 EU/mg) was procured from Yiyuan Biotechnology (Guangzhou, China). Masson’s trichrome stain and hematoxylin and eosin (H&E) staining kits were supplied by Solarbio. The following primary antibodies were used: anti-α-smooth muscle actin (α-SMA; cat #ab108531), anti-matrix metalloproteinase-9 (MMP-9; cat #ab142180), and anti-CD68 (cat #ab283654), all from Abcam (Cambridge, United Kingdom).

### Fabrication of platelet membrane-camouflaged EMP nanoplatform

α,ω-Dicarboxyl polyethylene glycol (HOOC-PEG-COOH) was synthesized via esterification of polyethylene glycol (Mw = 2000) with succinic anhydride (molar ratio 1:2) in anhydrous dichloromethane under a nitrogen atmosphere for 48 h at 25 °C. Poly (ε-caprolactone) (PCL, Mn = 5,000) was prepared by ring-opening polymerization of ε-caprolactone catalyzed by tin (II) 2-ethylhexanoate (Sn(Oct)_2_, 0.1 mol%) at 110 °C for 24 h under argon. The amphiphilic block copolymer HOOC-PCL-PEG was synthesized via a DCC/DMAP coupling reaction (molar ratio 1:1.2:1.2 for PEG-COOH/PCL-OH/DCC) in anhydrous DCM at 25 °C for 24 h (3-Hydroxypropyl) triphenylphosphine bromide (TPP-OH) was obtained by nucleophilic substitution of triphenylphosphine (1.2 eq) with 3-bromo-1-propanol (1 eq) in dry acetonitrile at 80 °C for 12 h. The mitochondrial-targeting copolymer TPP-PCL-PEG was prepared through DMAP/DCC-mediated esterification (TPP-OH:HOOC-PCL-PEG = 1.5:1) in anhydrous DMF at 25 °C for 48 h. EPPT nanoparticles were fabricated by solvent evaporation: a THF solution containing TPP-PCL-PEG/EMP (10:1 w/w) was dropwise (1 drop/s) injected into an aqueous phase under magnetic stirring, followed by THF removal.

Platelet-derived membranes (PDMs) were isolated from Sprague-Dawley rat blood through differential centrifugation (300 × g for 10 min, then 2,500 × g for 20 min at 4 °C) and purified by hypotonic lysis with 10 mM Tris-HCl (pH 7.4). PDM-coated nanoparticles (PM@EPPT) were prepared by incubating EPPT with PDMs (1:1 w/w protein ratio) at 37 °C for 1 h, followed by extrusion through 400 nm polycarbonate membranes (11 passages).

### Characterization of nanoparticles

^1^H NMR spectra were recorded on a Bruker AVANCE III HD spectrometer (500 MHz). FT-IR spectra were acquired using a Thermo Scientific Nicolet iS10 spectrometer. Hydrodynamic diameter and zeta potential were measured by dynamic light scattering (Malvern Panalytical Zetasizer Nano ZS). Morphology was characterized by transmission electron microscopy (FEI Tecnai G2 F20 S-TWIN, 200 kV). Drug loading and encapsulation efficiency were quantified via HPLC (Waters 1,525 system equipped with a UV/Vis detector, C18 column, and an acetonitrile/water mobile phase). *In vitro* release studies were performed in PBS (pH 7.4) at 37 °C. Hemocompatibility was assessed by a hemolysis assay using fresh Sprague-Dawley rat erythrocytes (3.7% w/v). Colloidal stability was evaluated by Tyndall scattering and DLS in DMEM containing 10% FBS at 37 °C.

### Cell culture

RAW 264.7 murine macrophages (ATCC^®^ TIB-71™, passages 5–15) were cultured in high-glucose DMEM containing 10% heat-inactivated FBS and 1% penicillin-streptomycin (100 U/mL penicillin and 100 μg/mL streptomycin). Cells were maintained at 37 °C with 5% CO_2_ and passaged at 80%–90% confluence using 0.25% trypsin-EDTA (Gibco, 25200056). All experiments used cells within 10 passages post thaw.

### Cell viability

Dose-dependent EMP cytotoxicity: RAW 264.7 cells (5 × 10^3^ cells/well in 96-well plates) were seeded and stabilized for 24 h, then treated with EMP (0–80 μM, 24 h). Cell viability was assessed by Cell Counting Kit-8 (CCK-8).

ox-LDL-induced foam cell formation model: Cells were pretreated with ox-LDL (80 μg/mL) for 24 h, followed by incubation with EMP (0–10 µM) for an additional 24 h before CCK-8 assay.

Nanoparticle biocompatibility assessment: Cells were incubated with PM@EPPT nanoparticles (0–400 μg/mL, 24 h) in serum-free DMEM. Viability was quantified by CCK-8 assay, with untreated cells as the negative control and 1% Triton X-100 as the positive control.

Live/dead cell staining: RAW 264.7 cells (2 × 10^4^ cells/well in glass-bottom confocal dishes) were treated with EPP, EPPT, or PM@EPPT (36 μg/mL, 24 h), stained with calcein-AM (2 µM) and PI (1.5 µM) for 30 min at 37 °C, and imaged by confocal laser scanning microscopy (CLSM; Olympus FV4000, 488 nm/561 nm excitation). This concentration is equivalent to 5 µM EMP, calculated based on a drug loading of 6.2%. Step 1: 5 µM EMP = 5 × 10^−6^ mol/L×450.52 g/mol = 0.0022526 g/L = 2.2526 μg/mL, Step 2: Concentration (NPs)= (2.2526 μg/mL)/0.062 = 36.33 μg/mL.

### Cellular internalization

RAW 264.7 cells were incubated with DiI-labeled nanoparticles (36 μg/mL) for 4 h, fixed with 4% paraformaldehyde (PFA, 10 min), washed with PBS three times, and counterstained with DAPI (2 μg/mL, 15 min) for nuclear staining. Cellular images were acquired using confocal laser scanning microscopy (CLSM; excitation/emission wavelengths: 559/599 nm for DiI, 405/461 nm for DAPI).

### Mitochondrial Localization

RAW 264.7 cells were incubated with DiI-labeled nanoparticles (36 μg/mL) for 4 h, fixed with 4% paraformaldehyde (PFA, 10 min), and stained with MitoTracker™ Green FM (30 min at 37 °C). Colocalization analysis was performed using Pearson’s correlation coefficient via the ImageJ Coloc2 plugin on CLSM images (488 nm/405 nm excitation wavelengths).

### ROS scavenging

RAW 264.7 cells were stimulated with lipopolysaccharide (LPS, 1 μg/mL) for 24 h, then treated with EMP (5 µM) or nanoparticles (36 μg/mL) for an additional 24 h. Cells were stained with CellROX™ Deep Red (5 μM, 20 min at 37 °C) and analyzed by flow cytometry (BD FACS Canto™ II, 640 nm excitation).

### Mitochondrial membrane potential (ΔΨ_m_)

RAW 264.7 cells were stimulated with lipopolysaccharide (LPS, 1 μg/mL) for 24 h, then treated with EMP (5 µM) or nanoparticles (36 μg/mL) for an additional 24 h. Cells were stained with JC-1 (5 μg/mL, 20 min) after treatments. ΔΨ_m_ was quantified by flow cytometry (FITC: 530/30 nm; PE: 585/42 nm) as the red/green fluorescence ratio.

### Verification of *in vivo* targeting

After 6 weeks of western-type diets, atherosclerosis in the ApoE^−/−^ mice was detected by ultra sound test. A volume of 200 μL of Cy5-labeled PP or PM@PPT was injected into the ApoE−/− mice via the tail vein. After 4h, the *ex vivo* imaging was captured using the CRI Maestro Imaging System (Cambridge Research and Instrumentation, Inc., United States of America).

### Apoptosis

RAW 264.7 cells were treated with ox-LDL (80 μg/mL) for 24 h, then incubated with EMP (5 µM) or nanoparticles (36 μg/mL) for an additional 24 h. Cells were stained with Annexin V-fluorescein isothiocyanate (Annexin V-FITC, 1:20 dilution) and propidium iodide (PI, 1 μg/mL) in binding buffer (10 mM HEPES-NaOH, pH 7.4, 140 mM NaCl, 2.5 mM CaCl_2_) for 15 min at room temperature in the dark. Apoptosis rates were analyzed by flow cytometry with the following quadrants: Q1: Annexin V^−^/PI^−^ (viable), Q2: Annexin V^+^/PI^−^ (early apoptotic), Q3: Annexin V^+^/PI^+^ (late apoptotic/necrotic).

### Animal model

Male ApoE^−/−^ mice (8-week-old) were housed under specific pathogen-free (SPF) conditions (22 °C ± 1 °C, 55% ± 5% humidity, 12-h light/dark cycle) and fed a Western-type diet (D12108C, Research Diets; 40% kcal fat, 1.25% cholesterol) for 12 weeks. Mice (n = 6 per group) received twice-weekly tail vein injections (100 µL) of PBS (vehicle), EMP (10 mg/kg (Hao et al., 2023)), or EMP-loaded nanoparticles (EPP/EPPT/PM@EPPT,161.3 mg/kg) for 8 weeks. Concentration (NPs)= (10 mg/kg)/0.062 = 161.3 mg/kg, 6.2% is the drug loading rate.

All procedures were approved by the North Sichuan Medical College (NSMC) Institutional Animal Care and Use Committee (IACUC, approval number: NSMC2025037) and performed in accordance with the NIH Guide for the Care and Use of Laboratory Animals.

#### Plaque quantification and histopathology

Aortic tissues from ApoE^−/−^ mice were dissected, fixed in 4% paraformaldehyde (PFA), and stained with 0.5% Oil Red O to quantify lipid deposition (ImageJ v1.53). Paraffin-embedded aortic root sections (5 µm) were immunostained with anti-CD68 (1:200 dilution) for macrophages, anti-α-SMA (1:500 dilution) for smooth muscle cells, and anti-MMP-9 (1:300 dilution) for protease expression. Digital images were acquired using a VS200 slide scanner (Olympus), and positive areas were quantified with ImageJ (threshold set at 2× background intensity).

#### Biochemical

Serum high-density lipoprotein cholesterol (HDL-C) and low-density lipoprotein cholesterol (LDL-C) levels were quantified using direct enzymatic assays (Roche Diagnostics, Mannheim, Germany) on a Cobas c501 analyzer. Triglycerides were measured via the GPO-PAP method (Wako, Osaka, Japan), with inter-assay coefficient of variation (CV) < 3%. All samples were analyzed in duplicate following National Cholesterol Education Program (NCEP) standardization protocols.

#### Safety profiling

Hematoxylin and eosin (HE)-stained tissue sections (5 µm thickness) of the heart, liver, spleen, lung, and kidney were histopathologically examined by a certified pathologist to evaluate structural abnormalities, including cellular necrosis, inflammation, and tissue degeneration.

### Statistical analysis

Statistical comparisons were performed using unpaired Student’s t-test for two groups, one-way analysis of variance (ANOVA) with Tukey’s *post hoc* test for multi-group comparisons. Data are expressed as mean ± standard deviation (SD). Statistical significance is denoted as: P > 0.05 (non-significant), P ≤ 0.05 (*), P ≤ 0.01 (**), P ≤ 0.001 (***), with significance set at P < 0.05. Analyses were conducted using GraphPad Prism v10.2.1 and SPSS v29.0.

## Results

### Construction and characterization of nanoparticles

**FIGURE 1:**
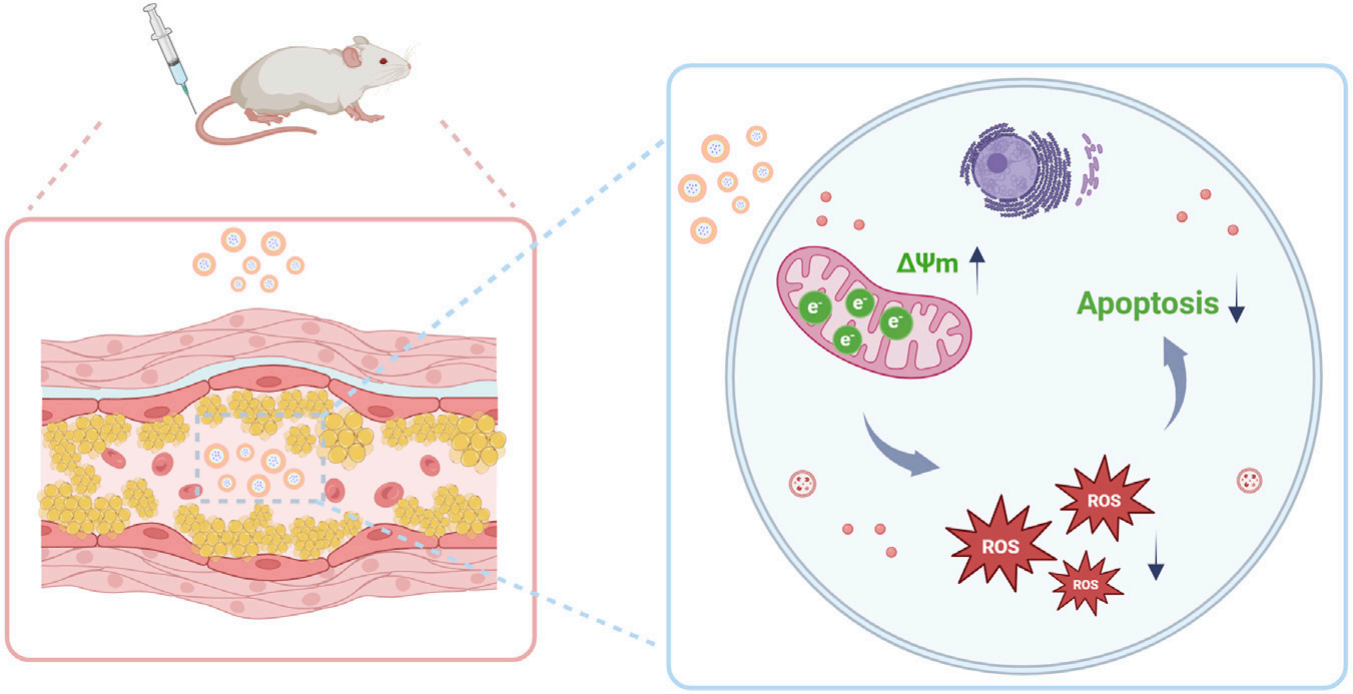
Schematic illustration of micelle-mediated EMP delivery for AS therapy. The engineered micelles, equipped with mitochondria-targeting capability, successfully delivered EMP to atherosclerotic plaques, subsequently restoring mitochondrial membrane potential (ΔΨ_m_), attenuating oxidative stress, and suppressing macrophage apoptosis.

**FIGURE 2:**
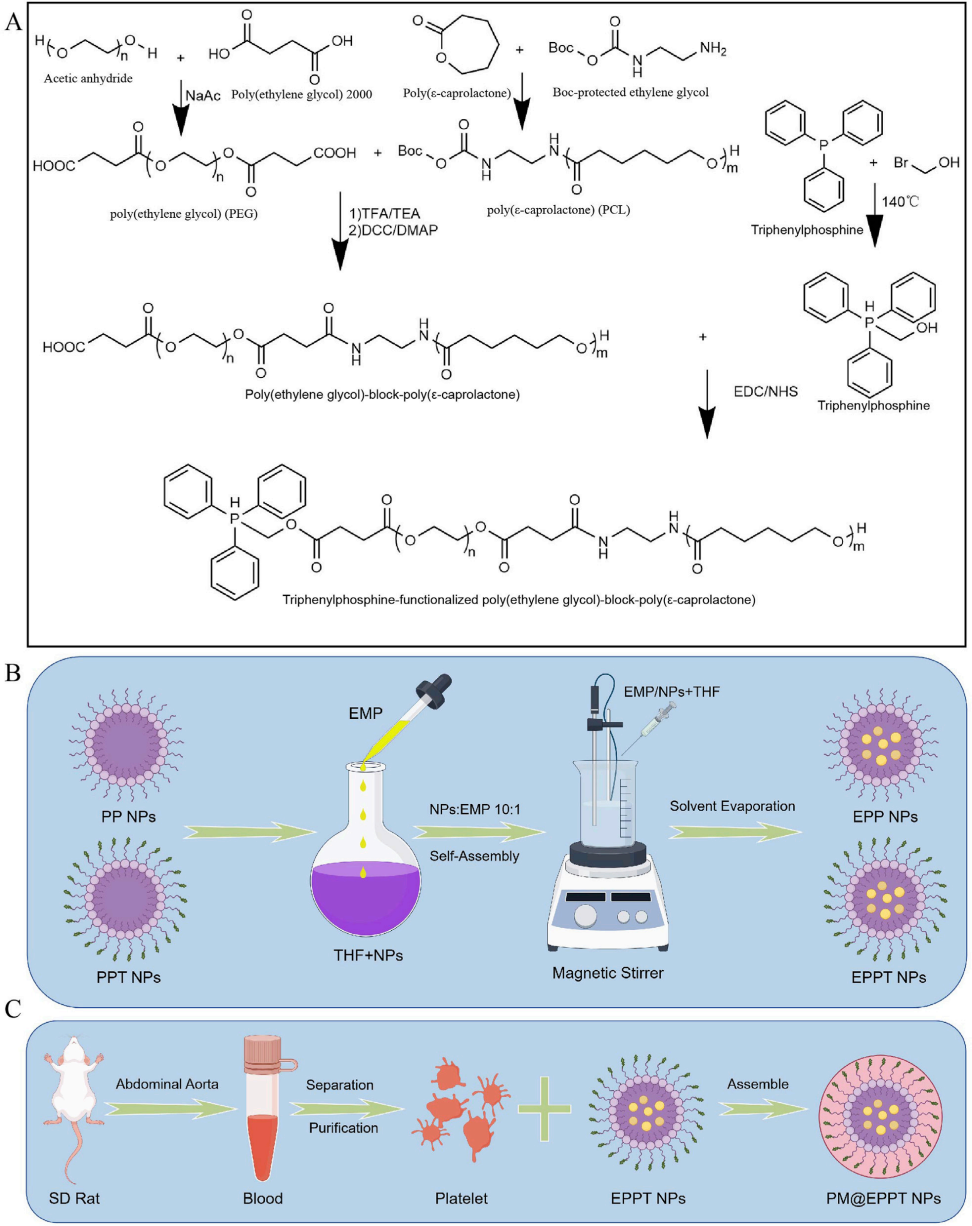
Nanomaterials Preparation. **(A)** Synthesis of PCL-PEG copolymers. Schematic of PCL-PEG (PP) and PCL-PEG-TPP (PPT) preparation via ring-opening polymerization of ε-caprolactone initiated by PEG. **(B)** Drug encapsulation. Empagliflozin (EMP) was loaded into nanoparticles (NPs) by the oil-in-water (O/W) solvent evaporation method (THF as the organic phase and 1% polyvinyl alcohol (PVA) as the stabilizer). **(C)** Platelet membrane coating. PM@EPPT was fabricated by coating EPPT NPs with isolated SD rat platelet membranes via ultracentrifugation at 3,000×g for 20 min at 4 °C. Figures B and C were created using Figdraw (ID: TAPSW1233a). Abbreviations: oil-in-water (O/W); poly (ethylene glycol)-poly (ε-caprolactone) (PCL-PEG); platelet membrane (PM); triphenylphosphine (TPP); tetrahydrofuran (THF).

**FIGURE 3:**
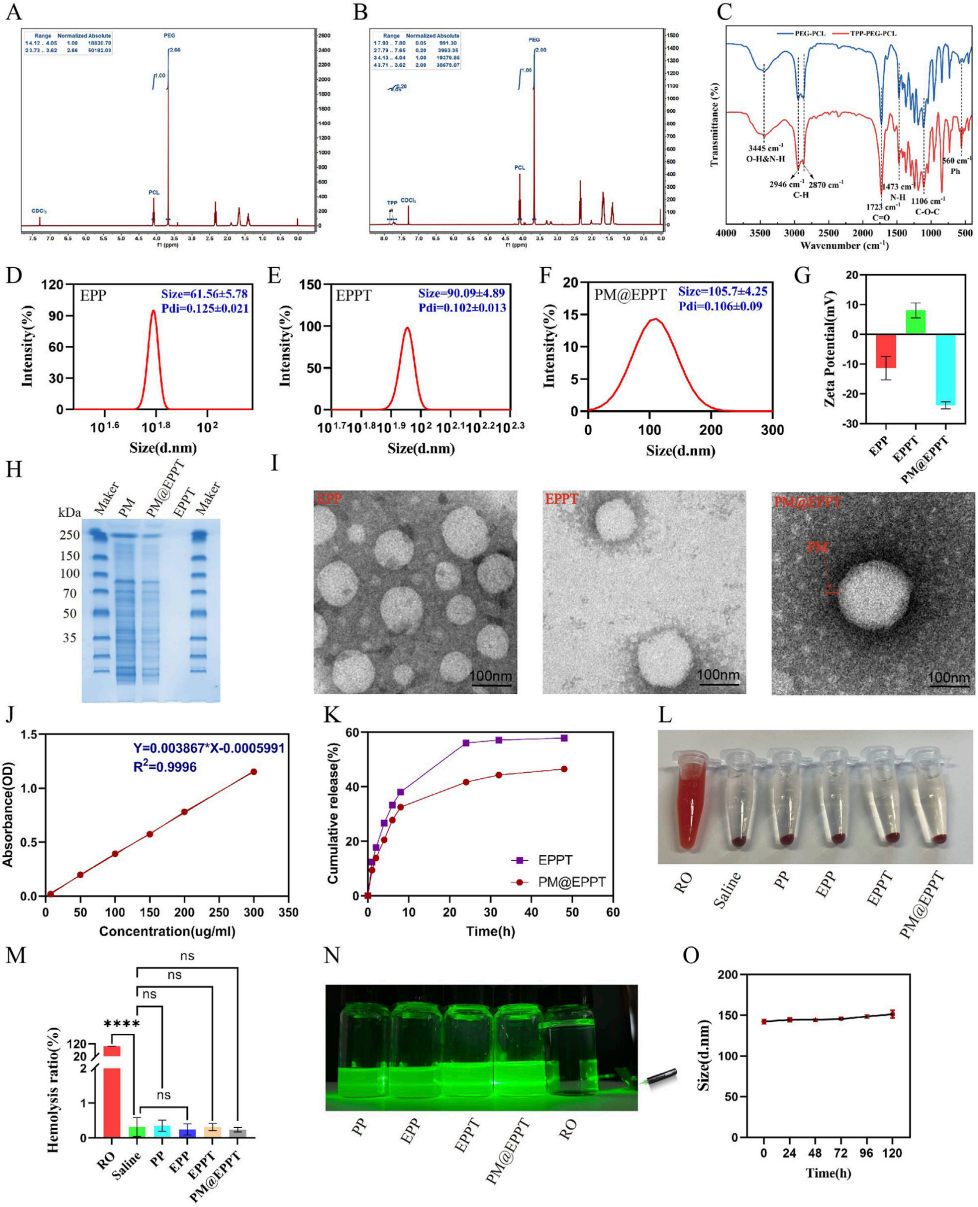
Characterization of PM@EPPT Nanoparticles (NPs). **(A,B)** ^1^H NMR spectra of PCL-PEG (PP) and PCL-PEG-TPP (PPT). **(C)** FTIR spectra of PP and PPT. **(D–F)** Particle size of EPP, EPPT, and PM@EPPT measured by dynamic light scattering (DLS). **(G)** Zeta potential distribution of EPP, EPPT, and PM@EPPT. **(H)** SDS-PAGE analysis of EPPT, platelet membrane (PM), and PM@EPPT. **(I)** Transmission electron microscopy (TEM) images of EPPT, PM, and PM@EPPT. **(J)** Standard curve of EMP measured by UV-Vis spectrophotometry at λmax = 254 nm. **(K)** Cumulative EMP release from free EPPT and PM@EPPT in saline (pH 7.4). **(L,M)** Hemolysis assay and quantitative analysis of RO (positive control), saline (negative control), and NPs. **(N)** Tyndall effect of RO, saline, and NPs. **(O)** Stability evaluation of PM@EPPT in DMEM +10% FBS over 5 days, monitored by size variation via DLS. Data are presented as mean ± standard deviation (SD, n = 3). Polydispersity index (PDI).

The PCL-PEG-TPP copolymer was synthesized via ring-opening polymerization of ε-caprolactone initiated by PEG_2000_, followed by TPP conjugation via nucleophilic substitution with 3-bromopropanol. EMP encapsulation was achieved by solvent evaporation, with subsequent platelet membrane functionalization to yield a bioinspired delivery platform (Figure 1 and Figures 2A–C). The ^1^H NMR spectrum of PCL-PEG exhibited characteristic peaks corresponding to PEG (δ 3.62 ppm, singlet) and PCL (δ 4.05 ppm, triplet), confirming successful copolymer synthesis. For PCL-PEG-TPP, additional aromatic proton peaks at δ 7.65–7.80 ppm (multiplet, 15H) were observed, verifying TPP conjugation (Figures 3A,B). FTIR analysis further supported successful synthesis: PCL-PEG displayed characteristic absorption bands at 3,445 cm^-1^ (O-H stretching), 2,946 cm^-1^ and 2,870 cm^-1^ (C-H stretching), 1723 cm^-1^ (C=O stretching), 1,473 cm^-1^ (CH_2_ bending), and 1,106 cm^-1^ (C-O-C stretching), while the distinct peak at 560 cm^-1^ in PCL-PEG-TPP was attributed to the P-Ph vibration of TPP (Figure 3C). DLS revealed hydrodynamic diameters of 61.56 ± 5.78 nm for EPP, 90.09 ± 4.89 nm for EPPT, and 105.3 ± 4.25 nm for PM@EPPT, with corresponding zeta potentials of −11.4 ± 3.9 mV, 8.1 ± 2.5 mV, and −23.9 ± 1.2 mV, respectively (Figures 3D–G). Sodium dodecyl sulfate-polyacrylamide gel electrophoresis (SDS-PAGE) demonstrated that PM@EPPT retained platelet membrane proteins, showing band patterns identical to native platelets (Figure 3H). TEM images showed uniformly dispersed spherical nanoparticles, with PM@EPPT exhibiting a characteristic dark outer ring corresponding to the platelet membrane coating, forming a clear core-shell structure (Figure 3I). HPLC quantification indicated PM@EPPT had a drug loading capacity of 6.2% and encapsulation efficiency of 87.0%. *In vitro* release studies demonstrated that the cumulative release of EPPT and PM@EPPT reached 57.8% and 41.7%, respectively, over 48 h at pH 7.4. Under identical conditions, PM@EPPT exhibited sustained-release behavior, likely due to encapsulation by platelet membranes which delayed drug liberation (Figures 3J,K). Hemocompatibility tests showed all nanoparticles induced negligible hemolysis (<5%) (Figures 3L,M). The Tyndall effect observed under laser irradiation demonstrated the stable colloidal dispersion of PM@EPPT. Furthermore, the nanoparticles exhibited excellent storage stability in 10% FBS-containing medium, with no significant size changes observed over 7 days (Figure 3O).

Cellular Interactions of PM@EPPT in RAW264.7 Macrophages: Cytotoxicity, Uptake Dynamics, Mitochondrial Localization, and Atherosclerotic Plaque Targeting.

**FIGURE 4:**
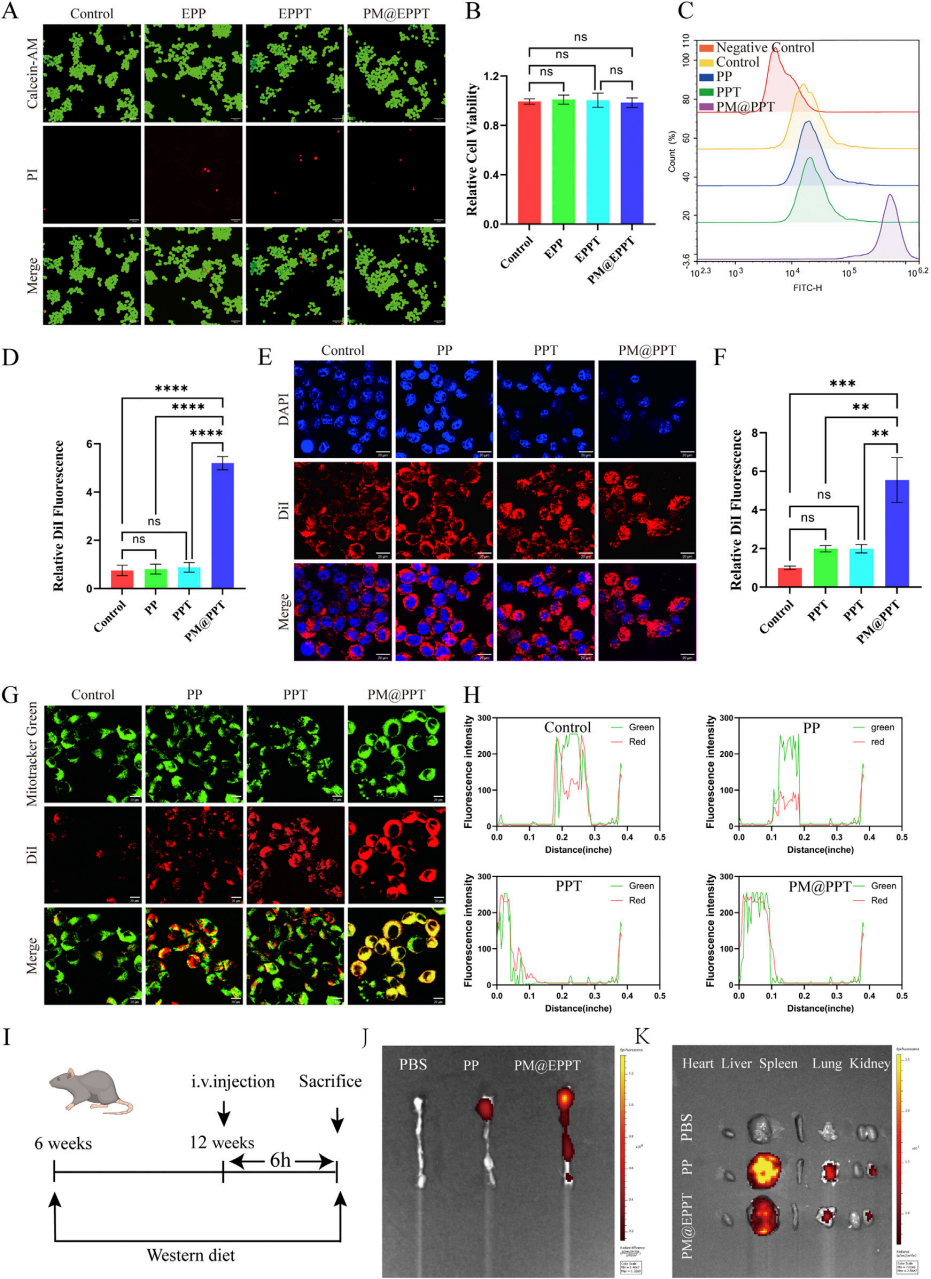
Cellular biosafety, cellular uptake, and mitochondrial localization study of NPs (PP, PPT, PM@EPPT). **(A)** Calcein-AM/PI staining (green = live; red = dead) of RAW 264.7 cells treated with NPs (36 μg/mL, 24 h) observed by confocal microscopy. Scale bar: 100 µm. **(B)** Corresponding quantitative analysis of fluorescence intensities in RAW 264.7 cells treated with NPs. **(C,D)** Cellular uptake study of RAW 264.7 cells treated with NPs (36 μg/mL, 4 h) analyzed by flow cytometry. **(E,F)** Cellular uptake study of RAW 264.7 cells treated with NPs imaged by confocal microscopy. **(G)** Mitochondrial localization of NPs (36 μg/mL, 4 h) in RAW 264.7 cells observed by confocal microscopy. Scale bar: 20 µm. **(H)** Quantitative analysis of mitochondrial localization in RAW 264.7 cells treated with NPs. **(I)** Experimental design scheme of nanoparticle targeting the ApoE^−/−^ mouse model. **(J,K)** After intravenous injection of the targeted nanoparticles, *in vivo* imaging showed that they specifically aggregated in the aortic arch plaques, heart, liver, spleen, lung and kidney regions (red signal). Data are presented as mean ± standard deviation (SD, n = 3).

As shown in Figures 4A,B, no significant cytotoxicity was observed for any tested material at a concentration of 36 μg/mL after 24 h of incubation. The cell viability values of EPP, EPPT, and PM@EPPT were 98.3% ± 2.1%, 97.6% ± 3.4%, and 99.2% ± 1.8%, respectively (vs. control 100% ± 1.9%), showing no statistically significant differences (P > 0.05). This confirms the absence of cytotoxicity from all nanoplatforms at the tested concentration (36 μg/mL). These findings establish critical concentration thresholds for subsequent therapeutic applications. Quantitative analysis of cellular uptake by flow cytometry (FCM; Figures 4C,D) revealed comparable DiI fluorescence intensity in PP (0.82% ± 0.40%, P > 0.05) and PPT (0.88% ± 0.40%, P > 0.05) groups versus Control (0.75% ± 0.43%). In contrast, PM@PPT exhibited significantly enhanced cellular accumulation (5.20% ± 0.56%), showing ∼6.9-fold higher fluorescence than Control (P < 0.001). TEM observations corroborated these findings: PPT (1.99 ± 0.41, P < 0.05), EPPT (1.99 ± 0.53, P < 0.05), and PM@PPT (5.55 ± 2.85, P < 0.001) all demonstrated significantly higher uptake relative to Control (1.00 ± 0.25) (Figures 4E,F). This pronounced uptake enhancement suggests that platelet membrane modification facilitates nanoparticle-macrophage membrane fusion. Mitochondrial colocalization studies using MitoTracker Green demonstrated progressively improved targeting efficiency: PP showed baseline localization, PPT exhibited moderate enhancement, while PM@EPPT displayed the most robust mitochondrial accumulation (Figures 4G,H), confirming the synergistic targeting effects of TPP modification and membrane coating. In order to check the ability of PPT and PM@PPT in targeting atherosclerotic plaques, Cy5-labeled PP and PM@PPT were intravenously injected into the atherosclerotic model mice. After 4 h, the aorta was removed, and the concentration of nanoparticles in the aorta was observed by fluorescence imaging. As shown in Figures 4I–K, almost no fluorescence signals were observed at the aortic arch in the PBS group, whereas significant fluorescence was determined in the Cy5-labeled PM@PPT group, demonstrating that PM@PPT did target the atherosclerotic plaque at the aortic arch.

### Multifunctional mitochondrial protection by PM@EPPT: restoration of membrane

#### Potential, Antioxidant Defense, Anti-Apoptotic Efficacy

**FIGURE 5:**
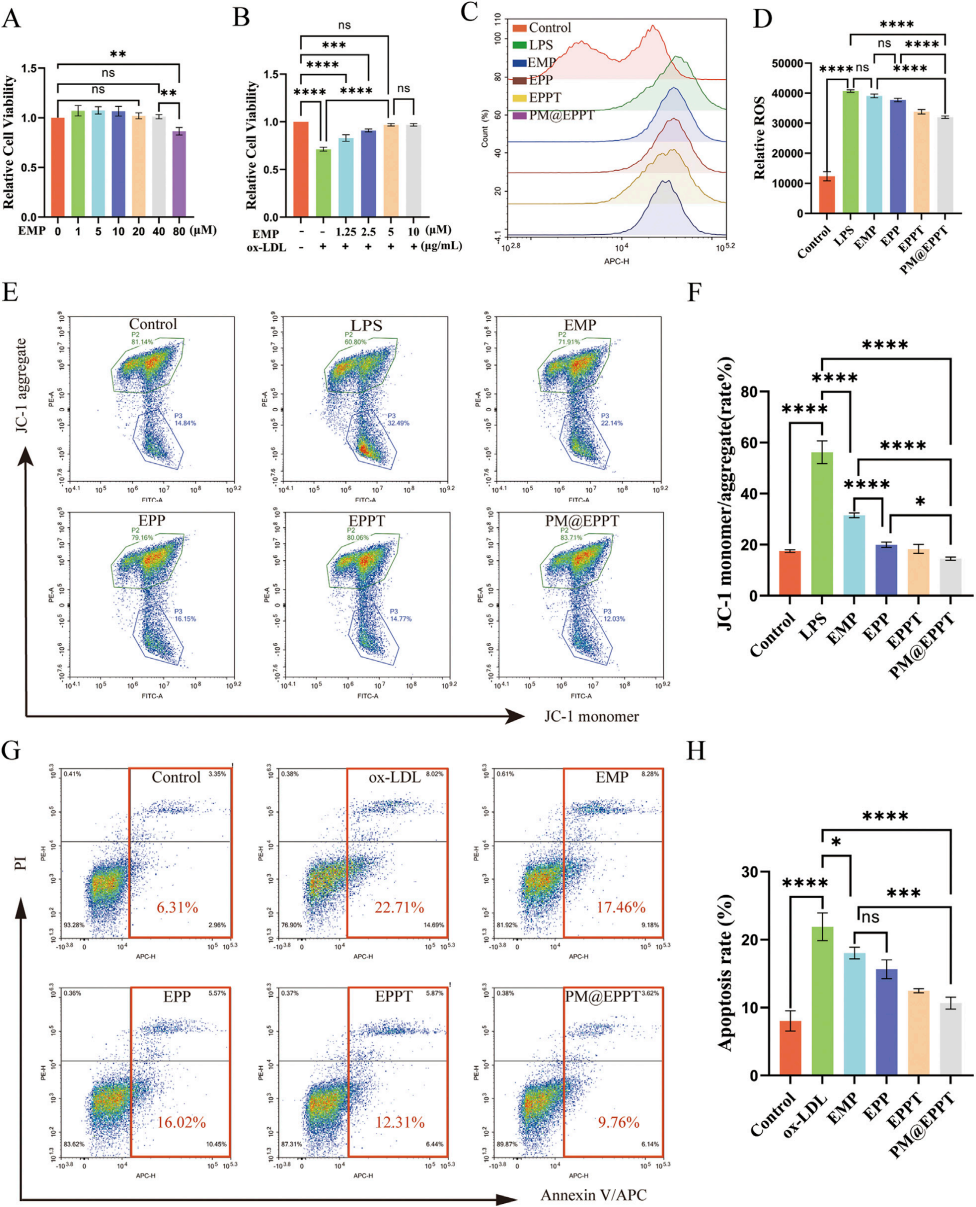
*In vitro* therapeutic effects of PM@EPPT. **(A)** Concentration-dependent cytotoxicity of EMP in RAW 264.7 cells (0–80 μM, 24 h, CCK-8 assay). **(B)** Dose optimization of EMP against ox-LDL (80 μg/mL)-induced cytotoxicity in RAW 264.7 cells. **(C,D)** Suppression of LPS-induced ROS generation by NPs (EMP, EPP, EPPT, and PM@EPPT) in RAW 264.7 cells. Cells were first stimulated with LPS (1 μg/mL) for 12 h, followed by pretreatment with NPs (36 μg/mL) for an additional 12 h. Intracellular ROS was detected using CellROX™ Deep Red. **(E,F)** NPs prevented LPS-induced loss of mitochondrial membrane potential in RAW 264.7 cells. Cells were treated with NPs (36 μg/mL) for 24 h, followed by LPS (1 μg/mL) stimulation for 12 h ΔΨ_m_ was measured by JC-1 fluorescence staining (excitation/emission: 488 nm/590 nm). **(G,H)** NPs attenuated ox-LDL-induced macrophage apoptosis. Cells were first stimulated with ox-LDL (80 μg/mL) for 12 h, then treated with NPs (36 μg/mL) for an additional 12 h. Apoptotic rates were determined by Annexin V/PI staining. Data are presented as mean ± standard deviation (SD, n = 3).

The CCK-8 assay demonstrated that EMP exhibited no significant cytotoxicity to RAW 264.7 cells at concentrations ranging from 0 to 40 μM, with cell viability remaining above 90% in all groups. Only marginal viability reduction (85% ± 3%) was observed at 80 µM (Figure 5A). In an ox-LDL-induced cytotoxicity model (80 μg/mL), EMP treatment significantly conferred concentration-dependent cytoprotection, with optimal efficacy achieved at 5 µM (Figure 5B). Oxidative stress represents a fundamental pathological mechanism in AS progression. CellROX™ Deep Red fluorescence analysis revealed that LPS stimulation significantly increased intracellular ROS levels (P < 0.001), while nanoparticle treatments effectively attenuated this oxidative stress. Notably, PM@EPPT demonstrated the most potent ROS-scavenging capacity (P < 0.001 versus EPP, EPPT, and LPS control groups) (Figures 5C,D). JC-1 probe staining confirmed mitochondrial membrane potential depolarization in LPS-treated cells, which was differentially restored by nanoparticle treatments. PM@EPPT exhibited superior ΔΨ_m_ preservation compared to EPP (P < 0.01) and EPPT (P < 0.05) (Figures 5E,F). Revealed apoptotic rate of 6.31% in normal controls, which increased significantly to 22.71% (P < 0.001) under ox-LDL stimulation. Treatment with EMP, EPP, EPPT, and PM@EPPT nanoparticles reduced apoptosis to 17.46% (P < 0.05), 16.02%, 12.31% and 9.76% (P < 0.001) respectively, with PM@EPPT restoring apoptosis to levels statistically indistinguishable from normal controls (P > 0.05) (Figures 5G,H).

#### PM@EPPT effectively inhibits the development of aortic atherosclerotic lesions in ApoE^−/−^ mice

**FIGURE 6:**
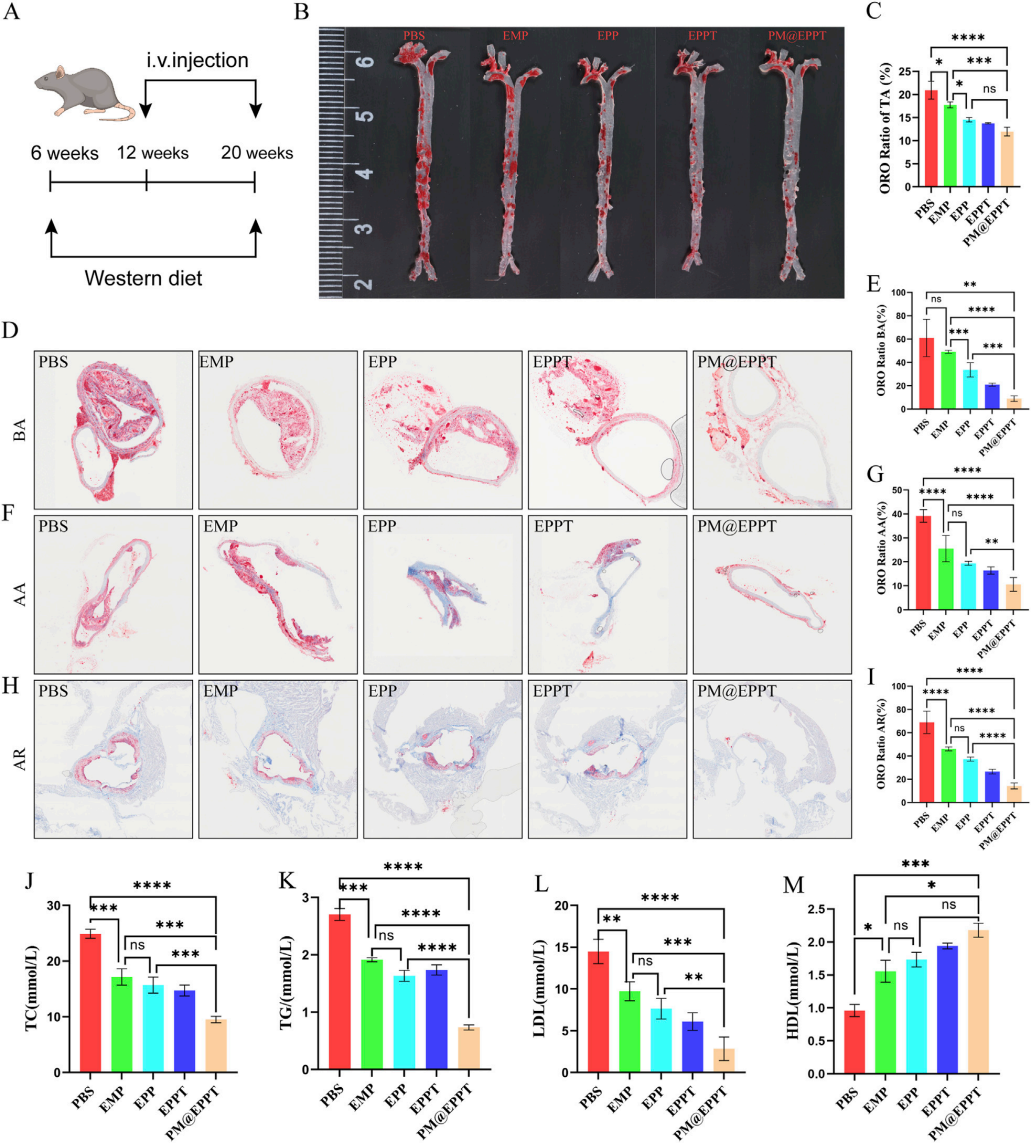
*In vivo* therapeutic efficacy of NPs against atherosclerotic plaque formation. **(A)** Experimental regime to evaluate therapeutic efficacy against atherosclerosis in ApoE^−/−^ mice. **(B,C)** Representative enface Oil Red O (ORO)-stained aortas after treatment with PBS, EMP, EPP, EPPT, or PM@EPPT *in vivo*. **(D,E)** Representative ORO images of the brachial artery and quantitative analysis of plaque area after treatment with PBS, EMP, EPP, EPPT, or PM@EPPT. **(F,G)** Representative ORO-stained images of the aortic arch after treatment with PBS, EMP, EPP, EPPT, or PM@EPPT. **(H,I)** Representative ORO-stained images of the aortic roots after treatment with PBS, EMP, EPP, EPPT, or PM@EPPT. **(J–M)** Serum concentrations of **(J)** total cholesterol (TC), **(K)** triglycerides (TG), **(L)** low-density lipoprotein (LDL), and **(M)** high-density lipoprotein (HDL)in mice following nanoparticle treatment. Data are presented as mean ± standard deviation (n = 6).

ApoE^−/−^ mice fed a high-fat diet were used to establish an AS model for evaluating the therapeutic efficacy of nanomaterials (Figure 6A). Oil Red O staining of aortic sections confirmed robust atherosclerotic plaque formation in PBS-treated control mice, validating successful model establishment (Figures 6B,C). In contrast, PM@EPPT treatment elicited a significant reduction in plaque burden. Quantitative regional analysis demonstrated a progressive decline in plaque area (%) across the treatment groups (PBS, EMP, EPP, EPPT, PM@EPPT) at key aortic sites: brachiocephalic artery (60.95%, 49.12%, 33.63%, 20.95%, 8.95%) (Figures 6D,E), aortic arch (39.16%, 25.52%, 19.41%, 16.37%, 10.58%) (Figures 6F,G), and aortic root (68.91%, 46.13%, 37.35%, 26.63%, 14.24%) (Figures 6H,I). PM@EPPT exhibited superior efficacy compared to the PBS control group (P < 0.001) and all other treatment groups (P < 0.01) across all vascular regions. *In vivo* blood analysis demonstrated that, compared to the control group, PM@EPPT effectively reduced total cholesterol (TC) (Figure 6J), triglycerides (TG) (Figure 6K), and low-density lipoprotein (LDL) (Figure 6L) (all P < 0.001), while increasing high-density lipoprotein (HDL) (Figure 6M), indicating potent efficacy in improving blood lipid profiles.

#### PM@EPPT Inhibits Atherosclerotic Lesions in ApoE^−/−^ Mice by Enhancing Plaque Stability and Systemic Biocompatibility

**FIGURE 7:**
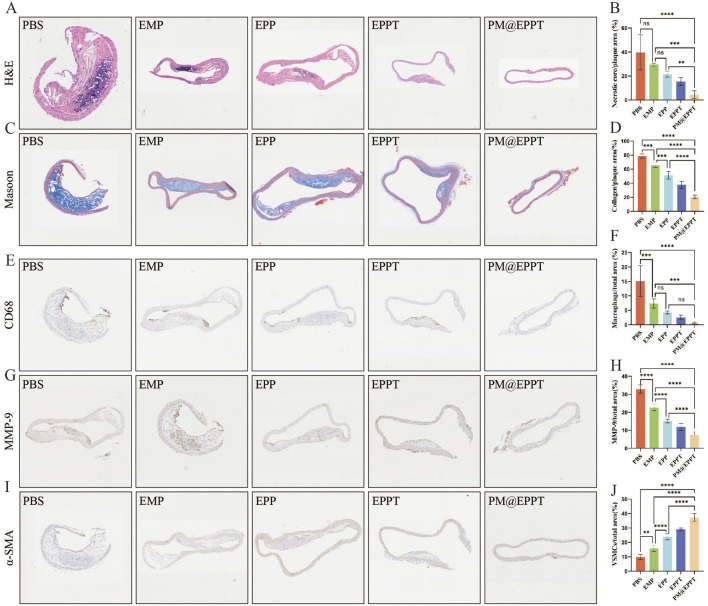
Multiplex immunohistochemical analysis of atherosclerotic lesions. **(A,B)** Representative hematoxylin and eosin (H&E) images of aortic roots and quantitative analysis of necrotic core area after treatment with PBS, EMP, EPP, EPPT, or PM@EPPT. **(C,D)** Representative images of collagen in the plaque areas stained by Masson’s trichrome. **(E–F)** Representative immunohistochemical images stained with anti-CD68 antibodies and quantitative data of the relative number of macrophages (cells/mm^2^) in plaque areas of the aortic root sections. **(G,H)** Representative immunohistochemical images stained with anti-MMP-9 antibodies and quantitative analysis of MMP-9 positive area percentage. **(I,J)** Representative immunohistochemical images stained with anti-α-SMA antibodies and quantitative data of smooth muscle cells (SMCs) in plaque areas of the aortic root sections. Data are presented as mean ± standard deviation (n = 6).

**FIGURE 8:**
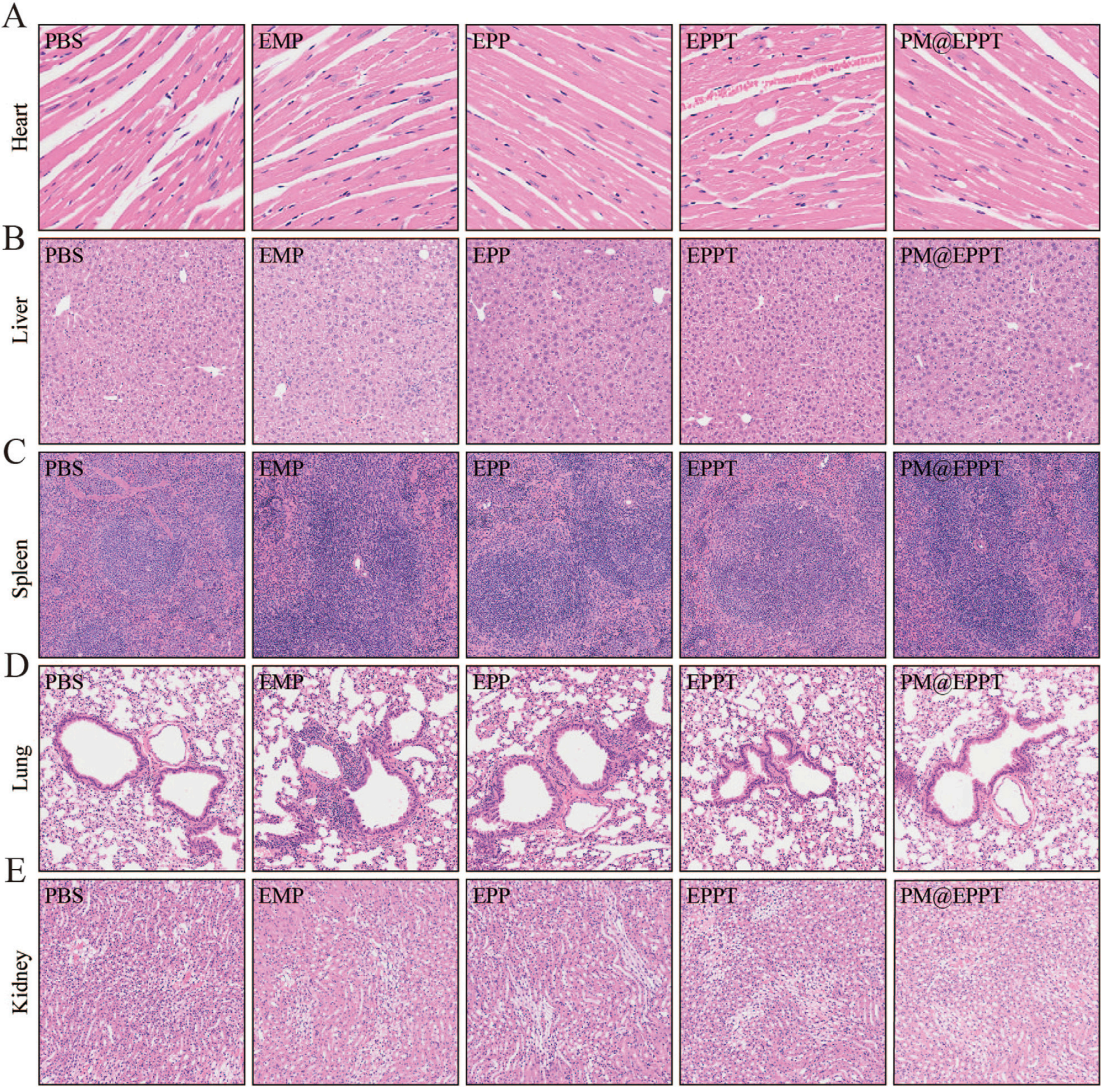
Histological observation of organs collected from ApoE^−/−^ mice after treatment. **(A–E)** Representative HE staining of heart, liver, spleen, kidney, and lung tissues after treatment with PBS, EMP, EPP, EPPT, or PM@EPPT.

Histopathological analysis further corroborated these findings. HE staining revealed a stepwise reduction in necrotic core area ratios (PBS: 39.77%, EMP: 29.51%, EPP: 21.62%, EPPT: 15.48%, PM@EPPT: 4.54%) (Figures 7A,B), while Masson’s trichrome staining indicated a progressive increase in collagen deposition (PBS:20.51%, EMP: 37.81%, EPP: 51.39%, EPPT: 65.69%, PM@EPPT:78.80%) (Figures 7C,D), reflecting enhanced plaque stability. Notably, PM@EPPT treatment conferred the most pronounced therapeutic benefits (P < 0.001). Immunohistochemical analysis further elucidated the mechanism underlying these effects. The PBS group exhibited pronounced macrophage infiltration (CD68-positive cells) and a concurrent reduction in vascular smooth muscle cells (α-SMA-positive cells), indicative of an unstable plaque phenotype. PM@EPPT treatment markedly suppressed CD68-positive cell infiltration while restoring α-SMA-positive cell content. Moreover, PM@EPPT significantly downregulated matrix metalloproteinase-9 (MMP-9) expression, suggesting its role in mitigating inflammation and extracellular matrix degradation (Figures 7E–J). Importantly, HE staining of major organs (heart, liver, spleen, lung, and kidney) demonstrated no detectable pathological alterations in nanoparticle-treated mice, underscoring the favorable biocompatibility and systemic biosafety of PM@EPPT (Figures 8A–J). Collectively, these findings highlight the therapeutic potential of PM@EPPT in AS management by suppressing lipid deposition, attenuating inflammatory responses, and enhancing plaque stability.

## Discussion

Given the multifactorial pathogenesis of AS and limitations of current therapies, developing novel mechanism-based treatments has become a research priority (Gallucci et al., 2024). Studies indicate that EMP exerts anti-AS effects through a dual mechanism: (1) Inhibiting the NF-κB pathway to mitigate vascular inflammation and promote collagen deposition, thereby delaying fibrosis (Liu Y. et al., 2021; Xu et al., 2024). (2) Activating the PINK1/Parkin pathway to enhance mitophagy and clear damaged mitochondria (Kloza et al., 2025; Yang et al., 2025). However, EMP’s low solubility, rapid clearance, and non-specific distribution lead to dose-dependent off-target toxicity, limiting its clinical translation (Biondi-Zocca et al., 2024; Hammad et al., 2024). To address this, we developed the PM@EPPT nanoparticles enhances drug accumulation at lesions and subcellular delivery precision, optimizing the therapeutic efficacy of EMP.

The PM@EPPT nanoparticles developed in this study comprises: A PCL-PEG copolymer core that encapsulates EMP via hydrophobic interactions to enhance solubility; Mitochondria-targeting TPP functionalization (Raza et al., 2024; Prakash, 2023; Tan et al., 2021; Verma et al., 2024; Sacchetti et al., 2024; Cheng et al., 2021). Which utilizes the ΔΨ_m_-driven electrochemical gradient to achieve precise subcellular localization; A platelet membrane biomimetic coating that prolongs circulation half-life and enhances lesion-specific accumulation through the natural affinity between adhesion molecules and lesion-associated DAMPs (Bruoha et al., 2024; Al Tahan and Al Tahan, 2024; Beerkens et al., 2025; Keethedeth and Anantha Shenoi, 2025). Physicochemical characterization revealed that PM@EPPT nanoparticles exhibited a hydrodynamic diameter of 105.83 ± 0.92 nm (PDI <0.2) and a zeta potential of −19.2 ± 0.3 mV. SDS-PAGE and SEM analyses confirmed complete coating with platelet membrane. *In vitro* and *in vivo* experiments have shown that PM@EPPT can target plaques and locate mitochondria in cells.

Among the existing developed systems, the platelet membrane-coated mesoporous silicon nanoparticles (PMSN) developed by Liang Chen et al. use inorganic mesoporous silicon as the carrier. However, they have slow degradation *in vivo*, are prone to deposition in organs such as the liver and spleen, and pose a risk of chronic toxicity (Chen et al., 2022). Moreover, their surface modification conditions are harsh, making it difficult to efficiently bind small molecule drugs or targeted ligands, and their functional expansion is limited. In contrast, the PCL-PEG carrier we adopted has both hydrophilicity and degradability. Among them, PCL can be hydrolyzed into small molecules through ester bonds and excreted. PEG can prolong circulation time, reduce clearance by the reticuloendothelial system (RES), and exhibits excellent biocompatibility. Furthermore, the PCL-PEG terminal functional groups can link small molecule drugs, targeted peptides, etc. Through mild reactions, significantly enhancing the intracellular targeting property of nanoparticles. Although the hyaluronic acid (HA) drug delivery system developed by Yizhou Wu et al. Treats AS with the combination of two drugs (pivastatin and proanthocyanidins), the nanomaterials use only hyaluronic acid as a single carrier and lack active targeting mechanism, resulting in limited drug enrichment efficiency at plaque sites. In contrast, our designed PM@EPPT nanoplatform combines the natural targeting function of platelet membranes with mitochondrial targeting properties, significantly enhancing drug enrichment concentration at plaque sites (Wu et al., 2025). Meanwhile, compared with the existing organic nanocarriers, The ST/NCP-PEG nanoparticles developed by Yuanzhe Lin et al. delivered lovastatin (Lin et al., 2024). We prepared PCL-PEG as the carrier by cross-linking and modifying PEG, which enhanced the affinity of the nanocell for hydrophobic drugs and improved the drug loading capacity. The PCL-SUCC nanoparticles developed by Emanuela F. Craparo et al. have not undergone structural modification and have relatively single functions (Craparo et al., 2020). We cross-linked PEG on the surface of nanoparticles and modified TPP (TPP is commonly used in the treatment of mitochondrial dysfunction diseases), which improved the hydrophilicity, circulation stability and targeting properties of the nanoparticles.

Given the pivotal roles of oxidative stress and apoptosis in AS pathogenesis (Li et al., 2025; Yan et al., 2024), we systematically elucidated PM@EPPT’s regulatory mechanisms on these pathological processes. Key findings include: LPS stimulation markedly increased intracellular ROS levels, which PM@EPPT treatment effectively reversed; JC-1 staining demonstrated PM@EPPT significantly ameliorated LPS-induced mitochondrial membrane potential depolarization; Flow cytometry analysis showed PM@EPPT reduced ox-LDL-induced apoptosis from 22.71% ± 2.13%–9.76% ± 1.05%. These results comprehensively demonstrate that PM@EPPT exerts anti-atherosclerotic effects through mechanisms including restoration of mitochondrial membrane potential, resistance to oxidative stress, and inhibition of cellular apoptosis.

In ApoE^−/−^ atherosclerotic model mice, PM@EPPT demonstrated remarkable therapeutic efficacy. Experimental results revealed that compared with the PBS control group, PM@EPPT treatment significantly reduced plaque area throughout the entire aorta, with particularly notable decreases in lipid deposition in the brachiocephalic artery, aortic arch, and aortic root regions (*P* < 0.001). The therapeutic effects were superior to those achieved with free EMP or other nanoparticle formulations. Histopathological analysis demonstrated that PM@EPPT effectively reduced necrotic core area while increasing collagen deposition, thereby enhancing plaque stability. Immunohistochemical assays showed that PM@EPPT significantly decreased CD68^+^ inflammatory cell infiltration and MMP-9 expression (Lo et al., 2024; Gerosa et al., 2023), while maintaining α-SMA + vascular smooth muscle cell populations (Elmarasi et al., 2024). Furthermore, PM@EPPT modulated lipid metabolism by reducing total cholesterol, triglyceride, and low-density lipoprotein levels, while increasing high-density lipoprotein concentration. These findings collectively demonstrate that PM@EPPT exerts its therapeutic effects through multiple mechanisms, including suppression of local inflammatory responses, improvement of plaque structure, and regulation of systemic metabolism, highlighting its potential as a promising therapeutic platform for AS intervention.

However, several key challenges remain to be addressed for the clinical translation of PM@EPPT: preparation of nanometers using approved human platelet membranes (Tikhonov et al., 2024; Fernández-Borbolla et al., 2024); Conduct a long-term safety assessment, including chronic toxicity, immunotoxicity and comprehensive pharmacokinetic analysis.

## Data Availability

The original contributions presented in the study are included in the article/supplementary material, further inquiries can be directed to the corresponding authors.

## Abbreviations

ApoE, Apolipoprotein E knockout; AS, Atherosclerosis; CD68, Cluster of Differentiation 68; CCK-8, Cell Counting Kit-8; DAPI, 4′,6-diamidino-2-phenylindole; DLS, Dynamic Light Scattering; EDTA, Ethylenediaminetetraacetic acid; H&E, Hematoxylin-eosin; HDL, High density lipoprotein; HPLC, High performance liquid chromatography; IR, Fourier transform infrared spectrometer; LDL, Low density lipoprotein; LPS, Lipopolysaccharide; MMP-9, Matrix metalloproteinase; ORO, Oil Red O; ox-LDL, Oxidized low density lipoprotein; PM, Platelet membrane; ROS, Reactive oxygen species; SDS-PAGE, Sodium dodecyl sulfate polyacrylamide gel electrophoresis; TC, Total cholesterol; TEM, Transmission electron microscope; TG, Triglycerides.

## References

[B1] Al Tahan M. A. Al Tahan S. (2024). Pioneering advances and innovative applications of mesoporous carriers for mitochondria-targeted therapeutics. Br. J. Biomed. Sci. 81, 13707. 10.3389/bjbs.2024.13707 39624468 PMC11608979

[B2] An Y. Ji C. Zhang H. Jiang Q. Maitz M. F. Pan J. (2025). Engineered cell membrane coating technologies for biomedical applications: from nanoscale to macroscale. ACS nano 19 (12), 11517–11546. 10.1021/acsnano.4c16280 40126356

[B3] Baaten C. Nagy M. Bergmeier W. Spronk H. M. H. van der Meijden P. E. J. (2024). Platelet biology and function: plaque erosion vs. rupture. Eur. heart J. 45 (1), 18–31. 10.1093/eurheartj/ehad720 37940193 PMC10757869

[B4] Beerkens A. P. M. Heskamp S. Reinema F. V. Adema G. J. Span P. N. Bussink J. (2025). Mitochondria targeting of oxidative phosphorylation inhibitors to alleviate hypoxia and enhance anticancer treatment efficacy. Clin. cancer Res. official J. Am. Assoc. Cancer Res. 31 (7), 1186–1193. 10.1158/1078-0432.ccr-24-3296 39898881

[B5] Behl A. Solanki S. Paswan S. K. Datta T. K. Saini A. K. Saini R. V. (2023). Biodegradable PEG-PCL nanoparticles for Co-delivery of MUC1 inhibitor and doxorubicin for the confinement of triple-negative breast cancer. J. Polym. Environ. 31 (3), 999–1018. 10.1007/s10924-022-02654-4 36405816 PMC9651876

[B6] Biondi-Zoccai G. Frati G. Peruzzi M. Booz G. W. (2024). Empagliflozin: primus inter pares among sodium-glucose Cotransporter-2 inhibitors? J. Cardiovasc. Pharmacol. 84 (3), 271–275. 10.1097/fjc.0000000000001605 39027982 PMC11973696

[B7] Björkegren J. L. M. Lusis A. J. (2022). Atherosclerosis: recent developments. Cell 185 (10), 1630–1645. 10.1016/j.cell.2022.04.004 35504280 PMC9119695

[B8] Bruoha S. Galli M. Sabouret P. Yosefy C. Taha L. Gragnano F. (2024). Atherosclerotic plaque erosion: mechanisms, clinical implications, and potential therapeutic Strategies-A review. J. Cardiovasc. Pharmacol. 83 (6), 547–556. 10.1097/fjc.0000000000001554 38421206

[B9] Chen L. Zhou Z. Hu C. Maitz M. F. Yang L. Luo R. (2022). Platelet membrane-coated nanocarriers targeting plaques to deliver Anti-CD47 antibody for atherosclerotic therapy. Research 2022, 9845459. 10.34133/2022/9845459 35118420 PMC8791388

[B10] Cheng B. Li J. Peng L. Wang Y. Sun L. He S. (2021). Efficacy and safety of restarting antiplatelet therapy for patients with spontaneous intracranial haemorrhage: a systematic review and meta-analysis. J. Clin. Pharm. Ther. 46 (4), 957–965. 10.1111/jcpt.13377 33537999

[B11] Chong B. Jayabaskaran J. Jauhari S. M. Chan S. P. Goh R. Kueh M. T. W. (2024). Global burden of cardiovascular diseases: projections from 2025 to 2050. Eur. J. Prev. Cardiol., zwae281. 10.1093/eurjpc/zwae281 39270739

[B12] Craparo E. F. Drago S. E. Giammona G. Cavallaro G. (2020). Production of polymeric micro- and nanostructures with tunable properties as pharmaceutical delivery systems. Polymer 200, 122596. 10.1016/j.polymer.2020.122596

[B13] Elmarasi M. Elmakaty I. Elsayed B. Elsayed A. Zein J. A. Boudaka A. (2024). Phenotypic switching of vascular smooth muscle cells in atherosclerosis, hypertension, and aortic dissection. J. Cell. physiology 239 (4), e31200. 10.1002/jcp.31200 38291732

[B14] Fernández-Borbolla A. García-Hevia L. Fanarraga M. L. (2024). Cell membrane-coated nanoparticles for precision medicine: a comprehensive review of coating techniques for tissue-specific therapeutics. Int. J. Mol. Sci. 25 (4), 2071. 10.3390/ijms25042071 38396747 PMC10889273

[B15] Fitchett D. Inzucchi S. E. Wanner C. Mattheus M. George J. T. Vedin O. (2020). Relationship between hypoglycaemia, cardiovascular outcomes, and empagliflozin treatment in the EMPA-REG OUTCOME® trial. Eur. heart J. 41 (2), 209–217. 10.1093/eurheartj/ehz621 31504427 PMC6945517

[B16] Gaborit B. Ancel P. Abdullah A. E. Maurice F. Abdesselam I. Calen A. (2021). Effect of empagliflozin on ectopic fat stores and myocardial energetics in type 2 diabetes: the EMPACEF study. Cardiovasc. Diabetol. 20 (1), 57. 10.1186/s12933-021-01237-2 33648515 PMC7919089

[B17] Gallucci G. Turazza F. M. Inno A. Canale M. L. Silvestris N. Farì R. (2024). Atherosclerosis and the bidirectional relationship between cancer and cardiovascular disease: from bench to bedside-part 1. Int. J. Mol. Sci. 25 (8), 4232. 10.3390/ijms25084232 38673815 PMC11049833

[B18] Gerosa C. Cerrone G. Suri J. S. Aimola V. Cau F. Coni P. (2023). The human carotid atherosclerotic plaque: an observational review of histological scoring systems. Eur. Rev. Med. Pharmacol. Sci. 27 (8), 3784–3792. 10.26355/eurrev_202304_32179 37140327

[B19] Hammad R. W. Sanad R. A. Abdelmalak N. S. Latif R. (2024). Cubosomal functionalized block copolymer platform for dual delivery of linagliptin and empagliflozin: recent advances in synergistic strategies for maximizing control of high-risk type II diabetes. Drug Deliv. Transl. Res. 14 (3), 678–695. 10.1007/s13346-023-01423-7 37805954 PMC10810935

[B20] Hao H. Li Z. Qiao S. Y. Qi Y. Xu X. Y. Si J. Y. (2023). Empagliflozin ameliorates atherosclerosis via regulating the intestinal flora. Atherosclerosis 371, 32–40. 10.1016/j.atherosclerosis.2023.03.011 36990029

[B21] Hernandez A. F. Udell J. A. Jones W. S. Anker S. D. Petrie M. C. Harrington J. (2024). Effect of empagliflozin on heart failure outcomes after acute myocardial infarction: insights from the EMPACT-MI trial. Circulation 149 (21), 1627–1638. 10.1161/circulationaha.124.069217 38581389 PMC11115458

[B22] Ijaz M. Aslam B. Hasan I. Ullah Z. Roy S. Guo B. (2024). Cell membrane-coated biomimetic nanomedicines: productive cancer theranostic tools. Biomaterials Sci. 12 (4), 863–895. 10.1039/d3bm01552a 38230669

[B23] Jan N. Bostanudin M. F. Moutraji S. A. Kremesh S. Kamal Z. Hanif M. F. (2024). Unleashing the biomimetic targeting potential of platelet-derived nanocarriers on atherosclerosis. Colloids surfaces. B, Biointerfaces 240, 113979. 10.1016/j.colsurfb.2024.113979 38823339

[B24] Jiang Y. Yu M. Song Z. F. Wei Z. Y. Huang J. Qian H. Y. (2024). Targeted delivery of mesenchymal stem cell-derived bioinspired exosome-mimetic nanovesicles with platelet membrane fusion for atherosclerotic treatment. Int. J. nanomedicine 19, 2553–2571. 10.2147/ijn.s452824 38505171 PMC10949310

[B25] Keethedeth N. Anantha Shenoi R. (2025). Mitochondria-targeted nanotherapeutics: a new frontier in neurodegenerative disease treatment. Mitochondrion 81, 102000. 10.1016/j.mito.2024.102000 39662651

[B26] Kloza M. Krzyżewska A. Kozłowska H. Budziak S. Baranowska-Kuczko M. (2025). Empagliflozin plays vasoprotective role in spontaneously hypertensive rats via activation of the SIRT1/AMPK pathway. Cells 14 (7), 507. 10.3390/cells14070507 40214461 PMC11987869

[B27] Kumric M. Urlic H. Bozic J. Vilovic M. Ticinovic Kurir T. Glavas D. (2023). Emerging therapies for the treatment of atherosclerotic cardiovascular disease: from bench to bedside. Int. J. Mol. Sci. 24 (9), 8062. 10.3390/ijms24098062 37175766 PMC10178593

[B28] Li L. Guo Z. Zhao Y. Liang C. Zheng W. Tian W. (2025). The impact of oxidative stress on abnormal lipid metabolism-mediated disease development. Archives Biochem. biophysics 766, 110348. 10.1016/j.abb.2025.110348 39961502

[B29] Lin Y. Liu J. Chong S. Y. Ting H. J. Tang X. Yang L. (2024). Dual-function nanoscale coordination polymer nanoparticles for targeted diagnosis and therapeutic delivery in atherosclerosis. Small Weinheim der Bergstrasse, Ger. 20 (47), e2401659. 10.1002/smll.202401659 39185808 PMC11579969

[B30] Liu Z. Ma X. Ilyas I. Zheng X. Luo S. Little P. J. (2021). Impact of sodium glucose cotransporter 2 (SGLT2) inhibitors on atherosclerosis: from pharmacology to pre-clinical and clinical therapeutics. Theranostics 11 (9), 4502–4515. 10.7150/thno.54498 33754074 PMC7977463

[B31] Liu Y. Y. Xu J. Wu M. Xu B. Kang L. (2021). Empagliflozin protects against atherosclerosis progression by modulating lipid profiles and sympathetic activity. Lipids health Dis. 20 (1), 5. 10.1186/s12944-021-01430-y 33436015 PMC7802233

[B32] Liu Q. Zou J. Chen Z. He W. Wu W. (2023). Current research trends of nanomedicines. Acta Pharm. Sin. B 13 (11), 4391–4416. 10.1016/j.apsb.2023.05.018 37969727 PMC10638504

[B33] Lorentzen L. G. Yeung K. Eldrup N. Eiberg J. P. Sillesen H. H. Davies M. J. (2024). Proteomic analysis of the extracellular matrix of human atherosclerotic plaques shows marked changes between plaque types. Matrix Biol. plus 21, 100141. 10.1016/j.mbplus.2024.100141 38292008 PMC10825564

[B34] Mahmood T. Sarfraz R. M. Ismail A. Ali M. Khan A. R. (2023). Pharmaceutical methods for enhancing the dissolution of poorly water-soluble drugs. Assay drug Dev. Technol. 21 (2), 65–79. 10.1089/adt.2022.119 36917562

[B35] Mao Y. Ren J. Yang L. (2023). Advances of nanomedicine in treatment of atherosclerosis and thrombosis. Environ. Res. 238 (Pt 2), 116637. 10.1016/j.envres.2023.116637 37482129

[B36] Martinez Bravo G. Annarapu G. Carmona E. Nawarskas J. Clark R. Novelli E. (2024). Platelets in thrombosis and atherosclerosis: a double-edged sword. Am. J. pathology 194 (9), 1608–1621. 10.1016/j.ajpath.2024.05.010 38885926 PMC11373056

[B37] Nardin M. Verdoia M. Cao D. Nardin S. Kedhi E. Galasso G. (2023). Platelets and the atherosclerotic process: an overview of new markers of platelet activation and reactivity, and their implications in primary and secondary prevention. J. Clin. Med. 12 (18), 6074. 10.3390/jcm12186074 37763014 PMC10531614

[B38] Nedkoff L. Briffa T. Zemedikun D. Herrington S. Wright F. L. (2023). Global trends in atherosclerotic cardiovascular disease. Clin. Ther. 45 (11), 1087–1091. 10.1016/j.clinthera.2023.09.020 37914585

[B39] Prakash S. (2023). Nano-based drug delivery system for therapeutics: a comprehensive review. Biomed. Phys. and Eng. express 9 (5), 052002. 10.1088/2057-1976/acedb2 37549657

[B40] Raza M. A. Sharma M. K. Nagori K. Jain P. Ghosh V. Gupta U. (2024). Recent trends on polycaprolactone as sustainable polymer-based drug delivery system in the treatment of cancer: biomedical applications and nanomedicine. Int. J. Pharm. 666, 124734. 10.1016/j.ijpharm.2024.124734 39343332

[B41] Sacchetti S. Puricelli C. Mennuni M. Zanotti V. Giacomini L. Giordano M. (2024). Research into new molecular mechanisms in thrombotic diseases paves the way for innovative therapeutic approaches. Int. J. Mol. Sci. 25 (5), 2523. 10.3390/ijms25052523 38473772 PMC10932156

[B42] Safdar A. Wang P. Muhaymin A. Nie G. Li S. (2024). From bench to bedside: platelet biomimetic nanoparticles as a promising carriers for personalized drug delivery. J. Control. release official J. Control. Release Soc. 373, 128–144. 10.1016/j.jconrel.2024.07.013 38977134

[B43] Singh D. (2024). Organelle targeted drug delivery: key challenges, recent advancements and therapeutic implications. Endocr. metabolic and immune Disord. drug targets 24 (13), 1480–1487. 10.2174/0118715303282573240112104035 38303531

[B44] Sreena R. Nathanael A. J. (2023). Biodegradable biopolymeric nanoparticles for biomedical applications-challenges and future outlook. Mater. Basel, Switz. 16 (6), 2364. 10.3390/ma16062364 36984244 PMC10058375

[B45] Tan T. Yang Q. Chen D. Zhao J. Xiang L. Feng J. (2021). Chondroitin sulfate-mediated albumin corona nanoparticles for the treatment of breast cancer. Asian J. Pharm. Sci. 16 (4), 508–518. 10.1016/j.ajps.2021.03.004 34703499 PMC8520051

[B46] Tikhonov A. Kachanov A. Yudaeva A. Danilik O. Ponomareva N. Karandashov I. (2024). Biomimetic nanoparticles for basic drug delivery. Pharmaceutics 16 (10), 1306. 10.3390/pharmaceutics16101306 39458635 PMC11510494

[B47] Verma V. S. Pandey A. Jha A. K. Badwaik H. K. R. Alexander A. Ajazuddin (2024). Polyethylene glycol–based polymer-drug conjugates: novel design and synthesis strategies for enhanced therapeutic efficacy and targeted drug delivery. Appl. Biochem. Biotechnol. 196 (10), 7325–7361. 10.1007/s12010-024-04895-6 38519751

[B48] Vollset S. E. Ababneh H. S. Abate Y. H. Abbafati C. Abbasgholizadeh R. Abbasian M. (2024). Burden of disease scenarios for 204 countries and territories, 2022-2050: a forecasting analysis for the global burden of disease study 2021. Lancet London, Engl. 403 (10440), 2204–2256. 10.1016/s0140-6736(24)00685-8 PMC11121021 38762325

[B49] Wu Y. Zhou H. Liu H. Hu J. Sun Y. Yan W. (2025). Pitavastatin-loaded procyanidins self-assembled nanoparticles alleviate advanced atherosclerosis via modulating macrophage efferocytosis and cholesterol efflux. Acta Pharm. Sin. B 15, 3305–3320. 10.1016/j.apsb.2024.08.006 40654339 PMC12254700

[B50] Xu H. Fu J. Tu Q. Shuai Q. Chen Y. Wu F. (2024). The SGLT2 inhibitor empagliflozin attenuates atherosclerosis progression by inducing autophagy. J. physiology Biochem. 80 (1), 27–39. 10.1007/s13105-023-00974-0 37792168

[B51] Yadav J. Ahsan F. Panda P. Mahmood T. Ansari V. A. Shamim A. (2024). Empagliflozin-A sodium glucose Co-transporter-2 inhibitor: overview ofits chemistry, pharmacology, and toxicology. Curr. diabetes Rev. 20 (10), e230124226010. 10.2174/0115733998271026231127051545 38265382

[B52] Yan R. Zhang X. Xu W. Li J. Sun Y. Cui S. (2024). ROS-induced endothelial dysfunction in the pathogenesis of atherosclerosis. Aging Dis. 16 (1), 250–268. 10.14336/AD.2024.0309 38502586 PMC11745424

[B53] Yang J. Ye W. Wang K. Wang A. Deng J. Chen G. (2025). Empagliflozin promotes skin flap survival by activating AMPK signaling pathway. Eur. J. Pharmacol. 987, 177207. 10.1016/j.ejphar.2024.177207 39694175

[B54] Zhang Y. Zhang Q. Li C. Zhou Z. Lei H. Liu M. (2024). Advances in cell membrane-based biomimetic nanodelivery systems for natural products. Drug Deliv. 31 (1), 2361169. 10.1080/10717544.2024.2361169 38828914 PMC11149581

[B55] Zinger A. (2023). Unleashing the potential of cell biomimetic nanoparticles: strategies and challenges in their design and fabrication for therapeutic applications. J. Control. release official J. Control. Release Soc. 358, 591–600. 10.1016/j.jconrel.2023.04.040 37146767

